# A multi-million-year natural experiment: Comparative genomics on a massive scale and its implications for human health

**DOI:** 10.1093/emph/eoae006

**Published:** 2024-04-03

**Authors:** Iker Rivas-González, Jenny Tung

**Affiliations:** Department of Primate Behavior and Evolution, Max Planck Institute for Evolutionary Anthropology, Leipzig, Germany; Department of Primate Behavior and Evolution, Max Planck Institute for Evolutionary Anthropology, Leipzig, Germany; Department of Evolutionary Anthropology, Duke University, Durham, NC, USA; Department of Biology, Duke University, Durham, NC, USA; Faculty of Life Sciences, Institute of Biology, Leipzig University, Leipzig, Germany

## Abstract

Improving the diversity and quality of genome assemblies for non-human mammals has been a long-standing goal of comparative genomics. The last year saw substantial progress towards this goal, including the release of genome alignments for 240 mammals and nearly half the primate order. These resources have increased our ability to identify evolutionarily constrained regions of the genome, and together strongly support the importance of these regions to biomedically relevant trait variation in humans. They also provide new strategies for identifying the genetic basis of changes unique to individual lineages, illustrating the value of evolutionary comparative approaches for understanding human health.

KEYWORDS: comparative genomics; natural selection; mammals; nonhuman primates; genome assembly; phylogenomics

In the children’s game of telephone, a phrase gets passed along a chain of participants, and, while some information might be preserved by the end, the original meaning is typically indecipherable by the final version. In contrast, proverbs that are passed from generation to generation are also often modified, but retain a fundamental core meaning. For example, the English saying ‘*a bird in the hand is worth two in the bush*’ parallels similar sayings in other languages: ‘*a bird in the hand is worth more than two in the sky*’ in Portuguese, more than ‘*ten in the sky*’ in Dutch, and more than ‘*a hundred in the sky*’ in Spanish. In German and some Slavic languages, the bird in the hand is a sparrow, and it is worth more than a pigeon on the roof, or a dove on the branch. Small modifications aside, all versions preserve the same structure and function: a guaranteed possession is more valuable than a potential, but uncertain, gain.

Genomes evolve like proverbs, not like games of telephone. Through millions of years of evolution, natural selection constrains some parts of the genome from changing, but not others. By pinpointing these regions, researchers can identify locations in the genome that have a core function and, by extension, are likely indispensable for development, physiology, or behavior. Thirty-five years ago, Tagle and colleagues first leveraged this intuition to show that DNA sequence alignments of distantly related species could help identify gene regulatory elements by highlighting stretches of sequence that remain relatively unchanged across species [1] (Fig. 1). This approach, known as phylogenetic footprinting, became a foundational tool for annotating genes and regulatory elements in the early days of genome sequencing and assembly [4]. Nevertheless, the effectiveness of phylogenetic footprinting relies on the diversity of available genomes. Inadequate sampling, whether because the sample is limited to closely related species or because it is sparsely representative of an evolutionary tree, reduces the power to detect conserved regions and increases the probability that regions that appear to be conserved in a small sample are in fact not of special evolutionary interest.

**Figure 1.**
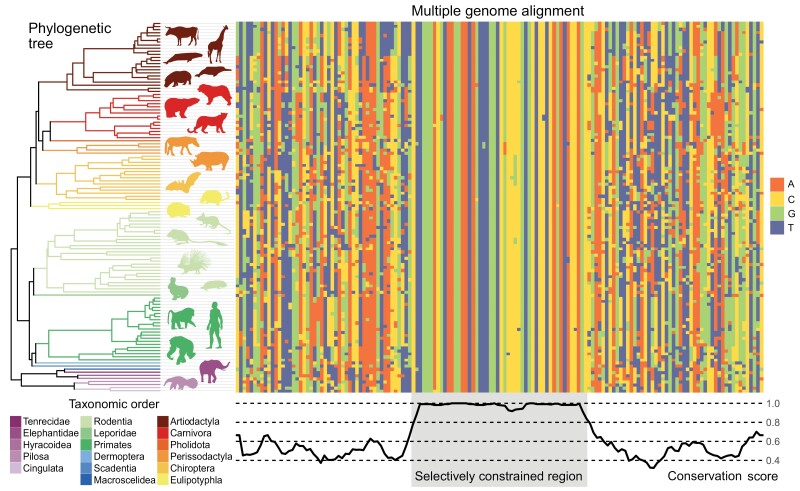
Identification of selectively important genomic regions using large-scale comparative genomics. Regions that have evolved unusually slowly in mammals are a product of selective constraint. The recent expansion of available genome assemblies confers new power to identify such regions. The logic behind these analyses is shown here, using the phylogeny of half of the mammalian species released as part of the Zoonomia Project (left) and an example multiple sequence alignment simulated using *msprime* [2] across the true phylogenetic tree. Each row of the central multiple sequence alignment corresponds to the simulated sequence at this locus for one species; each column corresponds to a single base pair in the sequence. A selectively constrained region appears in the center and is characterized by few to no changes across hundreds of millions of years of mammalian evolution (compared to neutral evolution in the flanking regions). The bottom trace shows per-base-pair conservation scores, calculated by subtracting the coefficient of unalikeability (an approach for estimating the variance of a categorical variable [3]) from 1. A conservation score of 1 corresponds to perfect conservation: the base observed in all aligned genomes is same. A value of 0 corresponds to the highest possible variance, where each of the four possible bases is observed 25% of the time. Note that this metric used here only for illustrative purposes; other conservation scores are typically used in practice for genomic analyses (e.g. phyloP scores, phastCons scores). The analyses presented in this figure could also be extended by adding multiple individuals per species. Selectively constrained regions would then show little within-species polymorphism compared to neutrally evolving regions, which would exhibit more within-species allelic variation.n

Last year, the number of available genome assemblies for primates and other mammals skyrocketed. In April, a special issue in *Science* marked the initial analysis of 240 mammalian genomes generated by the Zoonomia project [5]. In June, this achievement was followed by a second special issue reporting the initial analysis of 233 non-human primate genomes [6, 7]. Then, in November 2023, Kuderna et al. released a whole-genome alignment that includes 239 primates [8], nearly half of all extant primate species. Together, these sequences offer preliminary insight, as well as a set of remarkable resources, for investigating the last 200 million years of evolution in the mammalian branch of the tree of life. In addition to facilitating basic research in evolutionary, conservation, and population genetics, they also have four important implications for evolutionary medicine and research on human health.

First, such a dense sampling of extant genomes substantially increases the power to detect conserved regions across species, expanding the original application of phylogenetic footprinting to a massive scale (Fig. 1). Indeed, multiple contributions from both special issues focus on better estimating phylogenetic constraint to identify selectively important regulatory elements. Annotations resulting from these studies provide comprehensive, base-pair level assessments of constraint across mammals. They also reveal an interesting set of cases in which constraint is either stronger or only detectable, in primates relative to mammals more broadly. Many of these loci appear to have had biochemical functions prior to the expansion of primates but became more selectively important after our order arose [8]. Together, these contributions confirm that a substantial fraction of the mammalian genome is selectively constrained (3.3% of all bases at a false discovery rate of 5%, including 57.6% of coding sites [9]), and that, outside coding regions, constrained regions frequently overlap transcription factor binding sites, DNA hypersensitivity sites (i.e. ‘open chromatin’ regions), and other indicators of regulatory potential [8, 10].

Second, the recent wave of studies takes advantage of parallel progress in human genetics and functional genomics to directly investigate the degree to which constraint indicates regulatory function and/or differences in disease risk (notably, biochemically functional sequence is not always constrained [11]). For example, by integrating data from transgenic mice [8], massively parallel reporter assays [8, 12], and chromatin accessibility profiles [8, 13], these studies demonstrate that constrained sites are enriched in genes and regulatory elements active in the brain [8, 12, 13], including regions with the capacity to drive gene expression in primary cortical cells sampled in midgestation [13]. Meanwhile, by integrating evolutionary constraint metrics with results from genome-wide association studies (GWAS), Andrews et al. show that GWAS hits that overlap with highly constrained regions across mammals explain far greater (up to 20-fold more) trait heritability than expected by chance [10]. Sullivan et al. go one step further to show that base-pair level constraint scores can be directly integrated into GWAS analysis to improve fine-mapping (i.e. higher-resolution searches to pinpoint potential causal variants), including for health-relevant traits like body mass index and thyroid function [9]. Consequently, evolutionarily constrained regions not only have phylogenetic relevance for understanding mammalian evolution but also have outsized importance in explaining human phenotypic variation today. These results therefore emphasize the utility of comparative evolutionary approaches for identifying specific genes and pathways that account for trait heritability.

Third, several papers not only consider single reference genomes (as in classical phylogenetic footprinting) but also genetic polymorphism data within species. In doing so, they extend the original logic of phylogenetic constraint to shallower, population genetic timescales. The idea here is that variants that impose major fitness costs should not only be infrequent in cross-species comparisons but also appear at low allele frequencies within species. In support of this possibility, the integration of polymorphism data to highlight variants that are also rare in other primates (and hence likely selectively constrained) improves pathogenicity predictions for rare protein-coding variants in humans [14]. It also increases the overall predictive power of rare variant-based polygenic risk scores (i.e. composite summaries of disease risk based on genotypes across many variants) [15]. For example, people who carry a high burden of rare variants in lipid biosynthesis-related genes, particularly at loci where variation is unusual in other primates, represent a disproportionate fraction of clinically at-risk populations for diabetes and dyslipidemia [15]. Because of their low frequency, such variants are difficult to study using classical association approaches: they occur too infrequently to provide the statistical power for correlating genotype to phenotype. Data on genetic variation in other species therefore provide a complementary source of insight beyond what is possible in humans alone. Specifically, they both increase the resolution with which we can identify constrained sequences and contribute to the power of rare variant polygenetic risk scores—a class of variants that are otherwise very challenging to study.

Finally, identifying selectively constrained loci is of interest because it can also point to exceptions to the rule: cases in which a region has evolved under strong constraint through much of evolutionary history but has experienced unusually fast sequence turnover on one branch of the tree [12, 13]. Such a pattern can indicate a switch in evolutionary pressure from constraint to positive selection, and hence suggest changes that underlie the emergence of lineage-specific traits. This logic has already been used to highlight regions of the genome that may contribute to uniquely human traits [16]. For example, a developmental enhancer that regulates the gene *engrailed-1* is highly constrained in other primates but has evolved rapidly in the human lineage to alter the density of eccrine sweat glands in the skin [17]. However, the large set of recently released genome assemblies mean that the same procedure now can be applied in many more lineages. For instance, colobine monkeys—an Asian and African lineage about 30 million years diverged from our own—are the only primates that exhibit foregut fermentation, a dietary adaptation to folivory. By drawing on 49 of the highest-quality primate genome assemblies, Bi et al. showed that colobines also exhibit lineage-specific accelerated evolution in regions linked to metabolite detoxification and possibly maintenance of microbiota that help digest plant fiber [18]. As the number and quality of genome assemblies for vertebrates improve, this strategy may help resolve how other species have evolved traits outside the range observed in our own species—many of which are biomedically relevant (e.g. low cancer incidence in elephants and whales [19, 20], resistance to viral infection in bats [21], or extended longevity in Greenland sharks, which can live over 250 years [22]). Better assemblies—such as gapless telomere-to-telomere assemblies already coming online for humans [23] and a handful of other species—will also facilitate studies of constraint and adaptive evolution in structural variants (e.g. copy-number variants, indels, duplications, inversions, and translocations), which have known implications for disease risk and trait variation in humans [24] but cannot be effectively studied in most current-generation assemblies because of gaps in those sequences.

Together, the assemblies and resequencing data sets released in the past year thus provide an unprecedented view of mammalian biodiversity at the genetic level. They also contribute key evidence that identifying selectively relevant variation in our close living relatives overlaps with the mission of identifying genomic features relevant to human health. The next grand challenge for comparative genomics therefore lies in understanding the phenotypic impact of the candidate regions identified via sequence analysis—an undertaking that will require additional expertise in functional genomics, experimental animal models, and mammalian biology.
